# Effect of Anti-VEGF drugs on diabetic Retinopathy-Microaneurysms: A correlation study

**DOI:** 10.12669/pjms.40.11.9439

**Published:** 2024-12

**Authors:** Tianhang Li, Zhaohui Gu, Yueling Zhang, Jie Li, Juan Du, Yan Fu

**Affiliations:** 1 Tianhang Li, Ward of Ophthalmology 2nd Department, Baoding No.1 Central Hospital, Baoing 071000, Hebei, China; 2 Zhaohui Gu, Ward of Ophthalmology 2nd Department, Baoding No.1 Central Hospital, Baoing 071000, Hebei, China; 3 Yueling Zhang, Ward of Ophthalmology 2nd Department, Baoding No.1 Central Hospital, Baoing 071000, Hebei, China; 4 Jie Li, Ward of Ophthalmology 2nd Department, Baoding No.1 Central Hospital, Baoing 071000, Hebei, China; 5 Juan Du, Ward of Ophthalmology 2nd Department, Baoding No.1 Central Hospital, Baoing 071000, Hebei, China; 6 Yan Fu, Ward of Ophthalmology 2nd Department, Baoding No.1 Central Hospital, Baoing 071000, Hebei, China

**Keywords:** Diabetic retinopathy, Macular edema, Anti-vascular endothelial growth factor, Microaneurysm, Central retinal thickness, Best corrected visual acuity

## Abstract

**Objective:**

To investigate the efficacy of anti-vascular endothelial growth factor (VEGF) drugs in diabetic retinopathy (DR)-retinal microaneurysms, and its prognosis.

**Method:**

This was a retrospectively study in which total of 120 patients with DR in Baoding No.1 Central Hospital from June 2020 to June 2022 were retrospectively analyzed. According to different treatment methods based on macular edema, they were divided into an injection group and a control group. The control group was treated routinely, while the injection group was additionally intravitreally injected with an anti-VEGF drug. The patients were followed up for one year, and the changes in the number of retinal microaneurysms, best corrected visual acuity (BCVA) and central retinal thickness (CRT) were compared between the two groups. The effect on retinal microaneurysms was analyzed.

**Result:**

After treatment for 12 months, the total efficacy of the injection group was 95.00%, which was higher than 80.00% of the control group (*p<* 0.05). After one, three, six and twelve months of treatment, both CRT and the number of retinal microaneurysms reduced in the injection group compared with those before treatment. After treatment for one, three, six and twelve months, BCVA showed increases in the injection group, but no obvious changes in the control group compared with that before treatment.

**Conclusion:**

For patients with DR complicated with macular edema, early use of anti-VEGF drugs can significantly improve the fundus lesions, reduce the CRT and number of retinal microaneurysms, and improve the BCVA of the patients, with high clinical efficacy.

## INTRODUCTION

With the affluence of society, the prevalence of diabetes mellitus (DM) is increasing year by year. At present, more than 360 million people worldwide suffer from DM, of which more than 90% are type two diabetes mellitus (T2DM).^1^ It has been reported that about 1/3 of diabetic patients will develop diabetic retinopathy (DR)^2^, and the longer the course of DM, the higher the probability of secondary DR.^3^ Diabetic retinopathy (DR) is a common and severe complication caused by diabetic microangiopathy, which can lead to macular edema, macular ischemia, neovascular glaucoma (NVG) and vitreous hemorrhage. It mainly includes retinal microvascular injury and neurodegenerative diseases.^4^,^5^

At the early stage, retinal vascular endothelial cells are damaged, leading to vascular neoplastic changes and retinal microaneurysms. Due to not obvious initial clinical symptoms of DR, it can lead to impaired visual field, decreased visual acuity (VA) and night blindness without timely treatment, and even blindness in severe cases. At present, the main treatment methods for DR include drug therapy, vitrectomy, laser therapy and gene therapy.^6^ Patients with DR complicated with macular edema are mainly treated with drug therapy. Anti-vascular endothelial growth factor (VEGF) drugs can reduce the expression of VEGF and inhibit intraocular neovascularization.^7^ In addition, these drugs can be injected into the vitreous cavity to maximize their pharmacological effects, and have become the first-line drugs for diabetic macular edema. On this basis, the present study retrospectively analyzed anti-VEGF drugs in the treatment of DR, and evaluated the therapeutic efficacy and improvement in clinically relevant indicators, so as to provide a reference for anti-VEGF drugs in DR treatment.

## METHODS

This was a retrospective study. One-hundred and twenty patients confirmed with Diabetic Retinopathy (DR) treated in Baoding No.1 Central Hospital from June 2020 to June 2022 were divided into injection group (n=60) and control group (n=60) according to different treatment methods. The diagnostic criteria for DR referred to the 6-staging method for DR of the Ocular Fundus Disease Group, Ophthalmology Branch, Chinese Medical Association (CMA) in 2014.^8^

### Ethical Approval:

The study was approved by the Institutional Ethics Committee of Baoding No.1 Central Hospital (No.:2021076; date: May 27, 2021), and written informed consent was obtained from all participants.

### Inclusion criteria:

- Patients meeting the diagnostic criteria for T2DM, and presenting general condition suitable for fundus fluorescein angiography (FFA).
- Aged 60-79 years old.
- Patients without other fundus diseases except DR.
- Patients without history of intraocular surgery such as intravitreal drug injection, retinal laser photocoagulation, vitrectomy, etc.
- The injection group complicated with macular edema, and the control group not complicated with macular edema.
- Patients with complete medical records and auxiliary examination data.

### Exclusion criteria:

- Neovascular glaucoma (NVG).
- Vitreous hemorrhage/retinal neovascular membrane requiring vitrectomy.
- Refractive interstitial opacity such as lens opacity/walleye affecting fundus examination.
- Allergic constitution or abnormal renal function leading to an inability to FFA.
- Incomplete medical records and auxiliary examination data.

### Treatment methods:

The control group was given blood glucose control, calcium dobesilate for microcirculation improvement, and regular follow-up. On this basis, the injection group was additionally intravitreally injected with ranibizumab, an anti-VEGF drug. The surgical eyes were dripped with Levofloxacin Eye Drops three days before surgery, four times a day. During surgery, the patients were asked to lie flat on the operating table. After routine eye disinfection and towel laying, the eyelids were opened with an eyelid opener, the eyes were dripped with Proparacaine Hydrochloride Eye Drops for adequate anesthesia, and the conjunctival sac was cleaned with 5% povidone-iodine solution. One minute later, the eyes were cleaned with 500 ml of normal saline, and needled at 3.5-4.0 mm behind the limbus corneae perpendicular to the eyeball wall, with the tip slope facing the retina. Then, ranibizumab injection (0.05 ml) was slowly injected into the vitreous cavity. After removing the puncture needle, the injection point was gently pressed with a sterile cotton swab or sterile cotton ball for 2-3 minutes to prevent drug reflux. Subsequently, the surgical eyes were applied with Tobramycin and Dexamethasone Eye Ointment, and covered with sterile application. Postoperatively, the eyes were dripped with antibiotic eye drops for three d to prevent infection. The patients were followed up once a month after surgery, and followed up for 12 months. FFA and optical coherence tomography (OCT) were performed one, three, six and twelve months after surgery.

### Evaluating indicators:

The information of the included patients was collected from the Medical Record System of our hospital. The clinical efficacy was compared between the two groups according to the following efficacy criteria: effective: improvement in VA by more than two lines, absorption of most retinal hemorrhage, edema and exudation, and significantly disappeared neovascularization and basically disappearance of retinal leakage in FFA; stable: no changes in VA, no obvious changes in retinal hemorrhage, edema and exudation, and no significant changes of leakage in FFA; aggravated: aggravated retinal hemorrhage, edema and exudation, and increased leakage in FFA. Total effective rate = (effective cases + stable cases) /total cases × 100%. In addition, the changes in intraocular pressure (IOP), best corrected visual acuity (BCVA), central retinal thickness (CRT) and number of retinal microaneurysms were compared before treatment, one, three, six and twelve months after treatment. BCVA was measured with the International Standard Visual Acuity Chart. CRT was measured using ZEISS Angio OCT angiography. Retinal angiography was carried out using Heidelberg retinal angiography (HRA) (German). Retinal microaneurysms in the fixed area of the posterior pole were counted with Retmarker DR. CMT values were mainly measured and recorded during fundus examination.

### Statistical analysis:

The data were statistically analyzed using SPSS 22.0. According to the data of each indicator in the pre-survey, the sample size is estimated by 95% confidence interval, and the largest one is the sample size of the study. The sample size required for each group was ≥60 cases on the basis of Fisher exact probability. The measurement data were expressed as (*χ̅*±*S*). Their inter-group comparisons were conducted by the *t* test, and intra-group comparisons before and after treatment by the paired *t* test. The rates were compared with the c^2^ test. P*<* 0.05 was considered as statistically significant.

## RESULTS

There were 60 patients in the injection group and the control group, respectively. The injection group included 32 males and 28 females, aged 60-79 years, with an average age of (68.90 ± 5.20) years. In the control group, 34 males and 26 females were included, with an age of 60-80 years (average, 69.57 ± 5.26 years). No statistically significant differences were found in the basic data between the two groups, suggesting comparability (*p*> 0.05). Table-I.

**Table-I:** Comparison of general data between the two groups.

Item	Injection group (n = 30)	Control group (n = 30)	t/²	P
Age (year)	69.90±4.87	69.27±5.14	0.693	0.490
Gender (Male/Female, n)	32/28	34/26	0.135	0.0.714
VA before treatment	0.29±0.13	0.28±0.14	0.279	0.781
IOP (mmHg)	22.67±4.35	22.72±3.79	0.067	0.947

After treatment for 12 months, the symptoms in the two groups were improved to varying degrees. The total efficacy of the injection group was 95.00%, which was higher than 80.00% of the control group, indicating a statistically significant difference in clinical efficacy between the two groups (*p<* 0.05), Table-II.

**Table-II:** Comparison of clinical efficacy between the two groups after 12 months [n (%)].

Group	n	Effective	Stable	Aggravated	Total effective rate (%)
Injection group	60	51(50.00)	6(10.00)	3(5.00)	57(95.00)
Control group	60	36(42.50)	12(7.50)	12(20.00)	32(80.00)
*χ* ^2^					6.171
*p*					0.013

Before treatment, BCVA in the injection group was lower than that in the control group. After treatment for one, three, six and twelve months, BCVA showed no obvious changes in the control group, but increases in the injection group compared with that before treatment, with statistically significant differences (*p<* 0.05), Table-III.

**Table-III:** Comparison of BCVA between the two groups before and after treatment (*χ̅*±*S*).

Group	Before treatment	1 month after treatment	3 months after treatment	6 months after treatment	12 months after treatment
Injection group (n = 60)	0.23±0.10	0.37±0.1	0.43±0.12	0.54±0.12	0.73±0.11
Control group (n = 60)	0.22±0.11	0.33±0.1	0.38±0.11	0.48±0.11	0.67±0.10
*t*	0.773	2.164	2.579	2.606	2.931
*p*	0.441	0.032	0.011	0.010	0.004

Before treatment, CRT of the control group was thinner than that of the injection group. After one, three, six and twelve months of treatment, no obvious changes in CRT of the control group, but decreases in CRT of the injection group were found compared with that before treatment, with statistically significant differences (*p<* 0.05), Table-IV.

**Table-IV:** Comparison of CRT between the two groups before and after treatment (*χ̅*±*S*, μm).

Group	Before treatment	1 month after treatment	3 months after treatment	6 months after treatment	12 months after treatment
Injection group (n = 60)	465.60±43.15	356.45±39.63	320.85±40.25	300.68±39.11	200.92±35.48
Control group (n = 60)	240.89±34.53	241.24±31.03	241.15±30.08	242.21±28.92	242.32±28.21
*t*	31.498	17.729	12.286	9.312	7.074
*p*	0.000	0.000	0.000	0.000	0.000

There was no significant change in the number of microaneurysms per unit area in the control group before and after treatment. After treatment for one, three, six and twelve months, the number of microaneurysms presented no obvious changes in the control group, but reduced in the injection group compared with that before treatment, with statistically significant differences (*p<* 0.05), Table-V.

**Table-V:** Comparison in number of retinal microaneurysms between the two groups before and after treatment (*χ̅*±*S*).

Group	Before treatment	1 month after treatment	3 months after treatment	6 months after treatment	12 months after treatment
Injection group (n = 60)	18.33±3.23	16.28±3.17	14.17±2.85	11.15±1.86	9.18±1.26
Control group (n = 60)	19.13±2.09	19.30±2.10	20.23±2.04	20.35±1.74	20.15±1.63
*t*	1.610	6.146	13.386	28.032	41.215
*p*	0.110	0.000	0.000	0.000	0.000

## DISCUSSION

This study is a retrospective study, and the results showed that the efficacy of patients treated with anti VEGF drugs was significantly higher than that of patients treated with conventional drugs (P<0.05), indicating that the degree of retinal lesions in patients significantly improved after 12 months of anti VEGF drug treatment. It is generally believed that VEGF binds to VEGF receptors on retinal vascular endothelial cells, induces the formation of new blood vessels, 9,10 and destroys the blood retinal internal barrier, which plays a key role in the progress of diabetes retinopathy. Anti VEGF drugs can bind to VEGF receptors and inhibit the activity of VEGF. Several studies have confirmed that the use of anti VEGF drugs to treat diabetes retinopathy can significantly improve the vision of patients.^11^,^12^ Our research findings are consistent with multiple studies. Research has shown that anti VEGF drugs relax blood vessels by releasing nitric oxide, and ranibizumab can reduce neovascularization in the optic disc and eliminate macular edema, thereby reducing intraocular pressure in patients. The results of this study showed that after treatment with anti VEGF drugs, the intraocular pressure level of patients significantly decreased (P<0.05). The reason for this is that intravitreal injection of ranibizumab can directly act on the lesion, causing the drug to be located at a higher level in the short term, rapidly exerting drug action, reducing local inflammatory reactions, accelerating neovascularization, and other effects.^13^,^14^ In this study, the BCVA level of patients treated with anti VEGF drugs was significantly higher than that of the conventional treatment group (P<0.05), which is consistent with the results of multiple clinical studies.^15^-^17^ This indicates that this treatment regimen can effectively improve patients’ vision and enhance clinical efficacy. The reason for this is that ranibizumab can bind to VEGF antigen subtypes, leading to a decrease in their activity, a reduction in VEGF expression, obstruction of angiogenesis, delay of vascular exudation, control of ocular inflammatory response, alleviation of macular edema, and promotion of visual recovery in patients.^18^ Related literature shows that anti VEGF can reduce CRT.^19^,^20^ The results of this study showed that after treatment, the CRT of patients showed a significant decrease trend compared to before treatment, and the indicators were better than those of the control group (P<0.05), which is consistent with the results of related studies.^21^ The reason is that anti VEGF drugs can inhibit vascular leakage, regulate the blood retinal barrier, improve retinal blood circulation, promote subretinal blood absorption, reduce retinal edema, promote retinal function recovery, and ultimately reduce CRT.^22^

Microaneurysms are an important sign of diabetes retinopathy, and the pathogenesis of this vascular abnormality is still not very clear. Research shows that in patients with diabetes retinopathy,^23^ before diabetes retinopathy can be detected, eyeground microvessels have changed and neuroretina has been damaged, and most patients will miss the best treatment time. This study found that both groups of patients observed a decrease in the number of microaneurysms after treatment, and the decrease in the number of microaneurysms in patients was more significant after treatment with anti VEGF drugs (P<0.05). The mechanism may be that anti VEGF drugs bind to VEGF receptors on endothelial cells, causing endothelial cells that proliferate within microaneurysms to lose cytokine support and ultimately undergo apoptosis and degeneration.^24^,^25^

### Limitations:

It includes a few observed cases, short observation time and inadequate observation indicators. The clinical application value of anti-VEGF drugs in the treatment of DR needs to be further evaluated through enlarging the sample size, and increasing the follow-up duration and observation indicators.

## CONCLUSIONS

Early use of anti-VEGF drugs in patients with diabetic retinopathy can significantly improve the fundus lesions, decrease the IOP and CRT, reduce the number of retinal microaneurysms, and improve the BCVA of the patients, with high clinical efficacy.

### Authors’ Contributions:

**TL** and **ZG:** Carried out the studies, participated in collecting data, and drafted the manuscript, and are responsible and accountable for the accuracy or integrity of the work.

**YZ and JL:** Drafted the article, data analysis and revised it critically for important intellectual content.

**JD** and **YF:** Prepared the manuscript and did the statistical analysis, critical review.

All authors have read and approved the final manuscript.
